# The beneficial roles of apelin-13/APJ system in cerebral ischemia: Pathogenesis and therapeutic strategies

**DOI:** 10.3389/fphar.2022.903151

**Published:** 2022-08-10

**Authors:** Jiabin Li, Zhang Chen, Jingyu Chen, Yue Yu

**Affiliations:** ^1^ Department of Pharmacy, The Children’s Hospital, Zhejiang University School of Medicine, National Clinical Research Center for Child Health, Hangzhou, China; ^2^ Department of Tuina, The Third Affiliated Hospital of Zhejiang Chinese Medical University, Hangzhou, China; ^3^ Department of Critical Care Medicine, The Third Affiliated Hospital of Zhejiang Chinese Medical University, Hangzhou, China; ^4^ Department of Critical Care Medicine, Zhejiang Provincial People’s Hospital, People’s Hospital of Hangzhou Medical College, Hangzhou, China

**Keywords:** apelin-13, APJ, cerebral ischemia, pathway, angiogenesis, atherosclerotic plaque, excitotoxicity

## Abstract

The incidence of cerebral ischemia has increased in the past decades, and the high fatality and disability rates seriously affect human health. Apelin is a bioactive peptide and the ligand of the G protein-coupled receptor APJ. Both are ubiquitously expressed in the peripheral and central nervous systems, and regulate various physiological and pathological process in the cardiovascular, nervous and endocrine systems. Apelin-13 is one of the subtypes of apelin, and the apelin-13/APJ signaling pathway protects against cerebral ischemia by promoting angiogenesis, inhibiting excitotoxicity and stabilizing atherosclerotic plaques. In this review, we have discussed the role of apelin-13 in the regulation of cerebral ischemia and the underlying mechanisms, along with the therapeutic potential of the apelin-13/APJ signaling pathway in cerebral ischemia.

## 1 Introduction

Cerebral ischemia is a serious threat to human health, and is associated with high morbidity, disability and mortality. The rapidly aging population, as well as significant changes in lifestyle and diet brought about by the socio-economic development in China in recent years, has significantly increased the risk of stroke. It is currently the primary cause of death and disability among adults in China (Wang et al., 2015; Wang et al., 2017a; Guan et al., 2017). Therefore, it is crucial to devise suitable intervention methods in order to improve the prognosis of patients with cerebral ischemia and reduce the burden of disease.

Studies increasingly show that the apelin/apelin receptor (APJ) signaling pathway is involved in the occurrence and development of cerebral ischemia (Tables 1, 2). APJ is an orphan G protein-coupled receptor that was discovered by O'Dowd et al. (1993), and apelin is its endogenous ligand. The apelin/APJ system is ubiquitous in the peripheral and central nervous systems, and regulates blood pressure, myocardial contraction, immune response, angiogenesis, cancer development and other biological processes (Hosoya et al., 2000; Kawamata et al., 2001; Li et al., 2008; Barnes et al., 2010; Yang et al., 2016a). The currently known subtypes of apelin include apelin-12, apelin-13, apelin-17, apelin-28, and apelin-36, of which apelin-13 is the predominant subtype found in the heart, brain and hypothalamus. Previous studies have shown that apelin-13 plays an important role in cerebral ischemia (Duan et al., 2019; Wang et al., 2020) and ischemic stroke, and changes in the expression level of endogenous apelin-13 following ischemia has diagnostic and therapeutic relevance. Exogenous apelin-13 supplementation in ischemic stroke patients can play a neuroprotective role by regulating multiple signaling pathways. In this review, we have summarized the role and mechanism of apelin-13 in cerebral ischemia, in order to offer new insights into its diagnosis and treatment.

**TABLE 1:** The evidence from clinical trials demonstrating the role of apelin in stroke.

Subject	Main findings	Citation
68 MMD patients, 25 MCAO patients, 29 healthy controls	Apelin-13 is significantly increased in MMD patients than MCAO patients independent of NO and VEGF.	Wu et al. (2022)
60 patients with high risk of stroke (AF and non-AF group), 34 healthy controls	Apelin might be used to rule out AF in patients with high risk of stroke	Bohm et al. (2021)
109 AIS patients treated with intravenous thrombolysis	Apelin can help effectively forecast the occurrence of HT in AIS patients after intravenous thrombolysis, as an independent protective factor of HT.	Zhu et al. (2021)
156 ischemic stroke patients, 79 hemorrhagic stroke patients, 235 healthy controls	Higher vaspin, apelin, and visfatin levels might be associated with increased stroke risk	Yu et al. (2021)
244 AIS patients, 167 healthy controls	Serum apelin-13 may be a potential prognostic biomarker for AIS. Serum apelin-13 levels is lower in the patients than healthy controls, patients with a NIHSS score ≤3 had higher apelin-13 levels. There is an association between apelin-13 and death or major disability at the 3-months follow-up, the patients with high apelin-13 levels show a lower incidence of stroke and combined events at the 1-year follow-up	Wang et al. (2020)
168 AIS patients, 58 healthy controls	No difference of apelin between AIS patients and control group, and no difference of apelin between stroke subgroups with and without significant ipsilateral carotid stenosis	Kadoglou et al. (2014)

**TABLE 2:** The evidence from experimental trials demonstrating the role and mechanism of apelin in stroke.

Subject	Apelin treatment	Main findings	Citation
MCAO/R rats	Apelin-13 is injected into the tail vein 5 min before reperfusion	Apelin-13 attenuates injury following ischemic stroke by targeting MMP, endothelin-B receptor, occludin/claudin-5 and oxidative stress	Gholamzadeh et al. (2021a)
HT22 cells (OGD/R)	The cells are treated with 0.1 µM Apelin-36	Apelin-36 protects against OGD/R-induced oxidative stress and mitochondrial dysfunction by promoting SIRT1-mediated PINK1/Parkin-dependent mitophagy	Shao et al. (2021a)
Spragu-Dawley rats (MCAO/R), SH-SY5Y cells (OGD/R)	Apelin-13 (50 μg/kg) is injected into the right ventricle of rats at the onset of reperfusion; SH-SY5Y cell is treated with 10–7 M apelin-13 for 5 h	Apelin-13 inhibits apoptosis and excessive autophagy by upregulating Bcl-2 and activating mTOR signaling pathway after cerebral ischemia/reperfusion injury	Shao et al. (2021b)
Wistar rats (MCAO/R)	Intravenous injection of apelin-13 (10, 20, and 40 μg/kg) *via* tail vein 5 min before reperfusion	Apelin-13 improve sensory-motor balance defects by reducing neural death and infarct volume, and restoration of serum NO levels after cerebral ischemia	Gholamzadeh et al. (2021b)
Sprague-Dawley rats (SAH)	Apelin-13 (10 mg/kg) is injected into the lateral cerebral ventricle at 0.5 h after SAH.	Apelin-13 attenuates early brain injury following subarachnoid hemorrhage *via* suppressing neuronal apoptosis through the GLP-1R/PI3K/Akt signaling	Liu et al. (2019)
Sprague-Dawley rats (SAH)	Apelin-13 (25 μg/kg, 50 μg/kg, and 100 μg/kg) is injected intracerebroventricularly immediately after SAH induction	Apelin-13 attenuates early brain injury through inhibiting inflammation and apoptosis in rats after SAH.	Shen et al. (2022)
Sprague-Dawley rats (MCAO/R), PC12 cells (I/R)	Apelin-13 (30 μg/kg, 60 μg/kg, and 120 μg/kg) is injected intracerebroventricularly 15 min before reperfusion in rats; PC12 cells are pretreated with apelin-13 (0.5, 1, and 1.5 μM) for 6 h	Apelin 13 protects against I/R-induced ROS-mediated inflammation and oxidative stress through activating the AMPK/GSK-3β pathway *via* AR/Gα/PLC/IP3/CaMKK signaling, and further upregulates the expression of Nrf2-regulated antioxidant enzymes	Duan et al. (2019)
CD-1 mice (MCAO/R)	15 μl Apelin-12 is intracerebroventricularly injected 15 min before reperfusion	Apelin-12 inhibits the JNK and p38MAPK signaling pathway of the apoptosis-related MAPKs family, thus offering protection to neurons from ischemia-reperfusion injury	Liu et al. (2018)
118 MCAO patients and 22 controls patients; Sprague-Dawley rats (MCAO)	Pretreatment of apelin-17 (1 μmol/L) in rats	Plasma apelin-17 levels in ischemic stroke patients are positively associated with enhanced collateral circulation, which may have resulted from an apelin-17-induced cerebral artery dilation mediated through the NO-cGMP pathway	Jiang et al. (2019)
Wistar rats (MCAO/R)	Apelin-13 (10 μl) is injected intracerebroventricularly 30 min before MCAO in rats	Apelin-13 can attenuate activate neuronal apoptosis by inhibiting eIF2-ATF4-CHOP-mediated ER stress, involvement of Gαi/Gαq- CK2 signaling	Wu et al. (2018)
Wistar rats (MCAO/R)	10 µl apelin-13 (0.03 µg/µl) or10 µl apelin-36 (0.05 µg/µl) is injected into the right lateral ventricle at 2 h after MCAO.	Post-stroke administration of low-dose apelin-36 could attenuate infarct volume and apoptosis, which is associated with the inhibition of ERS/UPR activation. Low dose of apelin-13 had no protective effect in rats with ischemic stroke	Qiu et al. (2017)
AQP4 +/+ and AQP4 −/− mice (MCAO/R)	Apelin-13 (50 μg/kg) is injected intracerebroventricularly 15 min before reperfusion	Apelin-13 protects BBB from disruption after cerebral ischemia both morphologically and functionally, which is highly associated with the increased levels of AQP4, possibly through the activation of ERK and PI3K/Akt pathways	Chu et al. (2017)
Spragu-Dawley rats (MCAO/R); priamary neurons, astrocytes, and endothelial cells (OGD/R)	Apelin-13 (50 μg/kg) is injected intracerebroventricularly 15 min before or immediately after reperfusion in rats; the cells treat with apelin-13 (100 μmol/L)	Protective effects of apelin-13 on ischemic neurovascular unit injuries are highly associated with the increase of VEGF binding to VEGFR-2, possibly acting through activation of ERK and PI3K/Akt pathways	Huang et al. (2016)
Mice (MCAO/R)	Apelin-13 (100 μg/kg) is injected intracerebroventricularly 15 min before reperfusion	Apelin-13 protects against apoptosis by activating AMP-activated protein kinase pathway in ischemia stroke	Yang et al. (2016b)
C57/BL6 mice (BOCCA)	Intranasal administration of apelin-13 (4 mg/kg) is given 30 min after the onset of stroke and repeat once daily	Apelin-13 exert neuroprotective effect after ischemic stroke, through reducing inflammatory activities, decreasing cell death, and increasing angiogenesis	Chen et al. (2015)
Wistar rats (MCAO/R)	Apelin-13 (0.1 μg/g) diluted in 10 μl physiological saline is injected into the lateral ventricle	Apelin-13 is neuroprotective against cerebral ischemia/reperfusion injury through inhibition of neuronal apoptosis	Yan et al. (2015)
Wistar rats (MCAO/R)	Apelin-13 (50 ng/kg, 10 μl) is injected intracerebroventricularly at the onset of reperfusion	Apelin-13 is neuroprotective for neurons against I/R through inhibiting the neuroinflammation	Xin et al. (2015)
ICR mice (MCAO/R)	Apelin-13 (10 μg/kg, 50 μg/kg, 100 μg/kg, 5 μl) is injected intracerebroventricularly 15 min before reperfusion	Apelin-13 protects the brain against ischemia/reperfusion injury through activating PI3K/Akt and ERK1/2 signaling pathways	Yang et al. (2014b)
ICR mice (MCAO/R, H/I)	Apelin-36 (0.1 μg in 10 μl saline) is injected into the left lateral ventricle at 30 min before MCAO; apelin-36 (1 μg in 100 μl saline) is administrated intraperitoneally at the beginning of recovery (H/I)	Apelin-36 protects against ischemic brain injury by reducing apoptosis *via* activating the PI3K/Akt pathway	Gu et al. (2013)
Wistar rats (MCAO/R)	Apelin-13 (25, 50, and 100 μg in 5 μl saline) is injected intracerebroventriculary at the beginning of ischemia	Apelin-13 improves infarct volume, brain edema, and apoptosis, but not change neurological dysfunction after cerebral ischemia	Khaksari et al. (2012)
Primary mouse cortical neurons	Cortical neurons are incubated with different concentrations of apelin-13 (10 p.m. - 5 nM)	Apelin may block apoptosis and excitotoxic death *via* regulating Akt/ERK pathway and attenuating intracellular Ca^2+^ accumulation	Zeng et al. (2010)
Sprague-Dawley rats (SAH)	Apelin-13 (15 μg/kg, 50 μg/kg, and 150 μg/kg in 10 μl sterile saline) is injected intracerebroventricularly at 30 min after SAH induction	Exogenous apelin-13 binding to APJ attenuates early brain injury after SAH by reducing ERS-mediated oxidative stress and neuroinflammation, which is at least partly mediated by the AMPK/TXNIP/NLRP3 signaling pathway	Xu et al. (2019)
Sprague-Dawley rats (SAH)	Apelin-13 (15 μg/kg, 50 μg/kg, and 150 μg/kg in 10 μl sterile saline) is injected intracerebroventricularly at 30 min after SAH induction	Apelin-13 could exert its neuroprotective effects *via* suppression of ATF6/CHOP arm of ERS-response pathway in the early brain injury after SAH.	Xu et al. (2018)

## 2 Apelin-13

### 2.1 Biological characteristics of apelin-13

The gene encoding the arginine and lysin-rich apelin precursor peptide is located on chromosome Xg 25–26.1, and consists of three exons and two introns. The precursor peptide contains multiple potential sites of post-translational enzymatic processing, and can therefore generate multiple active apelin peptide fragments. For instance, cleavage of the apelin precursor peptide by angiotensin converting enzyme 2 (ACE2) generates isoforms of varying lengths such as aplein-12, apelin-13, apelin-17, and apelin-36. These isoforms differ in terms of tissue distribution, physiological and pharmacological effects, and binding strength with APJ (Lee et al., 2000; Reaux et al., 2001; De Mota et al., 2004). Furthermore, the biological activity of apelin, especially involving receptor binding and intracellular receptor transport, is greatly influenced by the size of the molecular fragment. Smaller apelin isoforms typically display stronger binding to APJ (Kleinz and Davenport, 2005; Carpéné et al., 2007).

Apelin-13 is a short peptide consisting of 13 amino acids. The N-terminal of apelin-13 binds to the APJ receptor, while the C-terminal is mainly involved in regulating its biological activity (Kawamata et al., 2001; Medhurst et al., 2003). It is degraded into an inactive form in the presence of ACE2 (Vickers et al., 2002), and is also modified into the more stable and active pyroglutamyl apelin-13. Studies show that pyroglutamyl apelin-13 is the most biological relevant subtype of apelin present in healthy human plasma (Mesmin et al., 2010; Zhen et al., 2013). Nevertheless, apelin-13 is ubiquitously expressed in the digestive system, cardiovascular system, central nervous system (CNS), kidneys, adipose tissue and retina, whereas apelin pro-peptide is predominantly present in the heart, lungs, kidneys and endothelial cells of large blood vessels (O'Carroll et al., 2000). Furthermore, apelin mRNA has been detected in the mammary glands, heart, lungs, brain, kidneys and other tissues of rats. Based on these findings, we can surmise that apelin has wide-ranging functions in humans as well as rodents. In particular, the presence of apelinergic neurons in the brain suggests that apelin may regulate food intake and digestion, pituitary hormone release and circadian rhythms (Reaux et al., 2002).

### 2.2 The APJ receptor

The apelin receptor APJ, also known as angiotensin II (Ang II) receptor-like 1, is a G protein-coupled receptor consisting of 380 amino acids with seven transmembrane structures. It is currently the only known apelin-13 receptor so far, and is highly expressed in neurons and glial cytoplasm in caudate nucleus, corpus callosum and hippocampus (Hosoya et al., 2000; Medhurst et al., 2003). APJ relays the signals through Gα subunit (Gαi or Gαq) of G protein. The structure of APJ is similar to that of the Ang II type I (AT1) receptor, although it cannot bind to Ang II (O'Dowd et al., 1993). In addition, G protein-independent signaling pathways are also involved in the activation of the apelin/APJ system. Upon binding to apelin, APJ is activated and recruits G protein-coupled receptor kinases (GRKs), resulting in APJ phosphorylation. The inhibitor protein (β-arrestin) then rapidly binds to APJ, resulting in receptor desensitization, and activation of the G protein-independent signaling pathways (Chen et al., 2014; Chen et al., 2020).

### 2.3 The tissue distribution pattern of apelin-13 and APJ

Apelin 13 is widely distributed in the CNS, with high expression levels in neurons and oligodendrocytes, and relatively lower expression in the astrocytes. Apelin 13 mRNA has been detected in the spinal cord, brain stem, cerebral cortex, hypothalamus, cerebellum, striatum, and hippocampus (O'Carroll et al., 2000). The differential expression pattern of apelin 13 and APJ in the CNS is indicative of multiple physiological or pathological functions. Both APJ and apelin are highly expressed in the hypothalamus, the master regulator of the neuroendocrine and humoral balance. The co-localization of apelin and hypothalamic arginine vasopressin (AVP) neurons suggests that apelin may regulate body fluid balance, feeding and drinking behavior and the HPA axis by interacting with AVP (De Mota et al., 2000; Reaux-Le Goazigo et al., 2004). In addition, the distribution of apelin in hypothalamus and pituitary region also indicates that apelin may be involved in the regulation of neurological and adenohypophysial hormones (Brailoiu et al., 2002; Yang et al., 2019).

Several studies have shown that apelin and APJ are highly expressed in the cardiovascular system, and can enhance myocardial contraction, reduce cardiac load, dilate blood vessels, promote angiogenesis, and regulate cardiac electrical conduction (Maguire et al., 2009; Aydin et al., 2014; Yu et al., 2014). Interestingly, the apelin/APJ system is also expressed in the cerebral blood vessels, and regulates vascular function. For instance, some studies have demonstrated that apelin can promote vasodilation in cerebral vessels (Nagano et al., 2019; Mughal et al., 2020). Mughal et al. (2018) found that apelin inhibits nitric oxide (NO)-dependent relaxation of cerebral arteries by activating APJ and inhibiting large-conductance, calcium-activated K channel in cerebral arterial smooth muscle cells, partially *via* a PI3K-dependent mechanism (Modgil et al., 2013). In addition, apelin promotes development of new blood branches from preexisting cerebral vessels following ischemic stroke (Han et al., 2015; Hiramatsu et al., 2017; Wu et al., 2017). Jiang et al. (2019) correlated the increased levels of plasma apelin-17 in ischemic stroke patients with enhanced collateral circulation, which can be attributed to cerebral artery dilation induced by apelin-17 *via* regulating the NO-cGMP pathway.

### 2.4 The neuroprotective effects of apelin 13

There is ample evidence demonstrating the neuroprotective effects of apelin-13. It can protect neuronal cells against apoptosis and excitotoxic injury by inhibiting NMDA-induced intracellular Ca^2+^ accumulation, oxidative stress, mitochondrial damage, cytochrome C release and caspase-3 activation *via* the ERK1/2 signaling pathway (Zeng et al., 2010). In addition, one study showed that supraspinal administration of apelin-13 in mice induced antinociception *via* the opioid receptor (Xu et al., 2009). The same group reported that apelin-13 relieved acetic acid-induced visceral pain in mice when injected into the subarachnoid space, and this analgesic effect was blocked by opioid receptor antagonists (Lv et al., 2012). Similarly, Hajimashhadi et al. (2017) demonstrated that intrathecal injection of apelin-13 increased the autonomic activity and relieved signs of pain in rats with spinal cord injury. However, one study showed that peripheral administration of apelin-13 reduced the latency of painful stimuli and enhanced pain sensitivity in a dose- and time-dependent manner (Canpolat et al., 2016), and intrathecal administration of ML221, an APJ antagonist, transiently reduced chronic constriction injury-induced pain hypersensitivity (Xiong et al., 2017). These findings suggest that the spinal apelin/APJ system may drive neuropathic pain. Thus, the regulatory effects of apelin-13 on pain may depend on the route of administration, as well as the type and degree of pain, and needs further clarification.

Previous studies have shown that apelin-13 can enhance the consolidation of passive avoidance learning and memory in mice, and these protective effects are neutralized by antagonists of α-adrenergic, cholinergic, dopamine, 5-hydroxytryptophan and γ -aminobutyric acid receptors, as well as inhibitors of nitric oxide synthesis (Telegdy et al., 2013). In a mouse model of chronic stress-induced memory deficit, apelin-13 significantly improved the cognition of new objects and memory deficit of Y maze, likely through to the upregulation of BDNF (Shen et al., 2019). In addition, exogenous apelin-13 attenuated cisplatin-induced cognitive dysfunction by activating the BDNF/TrkB signaling pathway and suppressing neuroinflammation. Apelin-13 is also known to relieve the symptoms of anxiety in mice, and these anti-anxiety effects may be related to α, *ß* adrenergic, dopamine and 5-HT receptors since they were blocked by the administration of phenbenzamine, haloperidol, propranolol, and dimethylergometrine (Telegdy and Jászberényi, 2014). Apelin-13 also reversed depression-like behavior in rats subjected to chronic social defeat stress and chronic water immersion restraint stress by regulating microglial polarization, and ameliorating a dysfunctional HPA axis and hippocampal glucocorticoid receptor (Dai et al., 2018; Tian et al., 2018; Zhou et al., 2020).

A clinical study on 126 patients with severe TBI and 126 healthy controls found that lower serum level of apelin-13 in the patients correlated significantly with increased severity of TBI, and was an independent predictor of short-term mortality, indicating that serum apelin-13 is a promising prognostic biomarker for severe TBI (Zhuang et al., 2021). The protective effects of apelin-13 in TBI are associated with inhibition of autophagy (Bao et al., 2015), suppression of neuronal apoptosis through the GLP-1R/PI3K/Akt signaling (Liu et al., 2019), and mitigation of blood-brain barrier (BBB) destruction and brain edema (Bao et al., 2016a). Early brain injury (EBI) is at present considered to the key determinant of the neurological function and clinical outcomes of subarachnoid hemorrhage (SAH) (Sehba et al., 2012; Fujii et al., 2013). Apelin-13 can attenuate EBI by inhibiting neuronal apoptosis and degeneration, and reducing the release of inflammatory cytokines such as TNF-α and IL-1β in the CSF. These protective effects were neutralized upon administration of the APJ inhibitor ML221 (Shen et al., 2022). The anti-apoptosis effect of apelin-13 in SAH may be related to the activation of the GLP-1R/PI3K/Akt signaling pathway (Liu et al., 2019). Xu et al. (2019) found that exogenous apelin-13 can alleviate EBI by suppressing endoplasmic reticulum (ER) stress-induced NLRP3 inflammasome activation and oxidative stress after SAH. Furthermore, the APJ inhibitor dorsomorphine reversed the neuroprotective effects of apelin-13 in SAH. Another study by Xu et al. (2018) confirmed that apelin-13 reduced neuronal apoptosis and prevented BBB disruption after SAH, and eventually improved EBI by alleviating ER stress partly *via* the ATF6/CHOP pathway. Intracerebral hemorrhage (ICH) shares certain pathological characteristics with SAH. Intracerebroventricular administration of apelin-13 improved motor function and brain edema after ICH by reducing neuronal death, which demonstrates its therapeutic potential (Bao et al., 2016b).

## 3 Apelin-13 and cerebral ischemia

The apelin/APJ system is closely associated with the pathogenesis of ischemic stroke, which is currently the most common cerebrovascular disease. Clinical studies suggest that apelin is related to the diagnosis and prognosis of cerebral ischemia, while studies in animal and cellular models indicate that exogenous apelin-13 can effectively reduce infarct volume and cerebral edema, and improve neurological function after cerebral ischemia.

### 3.1 Clinical studies

In a follow-up cohort study, Wang et al. (2017b) found that the variant rs9943582 of APJ gene was not significantly associated with ischemic stroke in the Chinese Han population. Consistent with this finding, another clinical study reported that the variant rs9943582 was not associated with the age at onset and clinical outcomes of ischemic stroke (Zhang et al., 2017). However, other clinical studies have reported contradictory findings. One study conducted in China on 244 AIS patients recruited within 24 h of stroke onset and 167 healthy controls showed that serum apelin-13 levels were lower in the patients compared to the healthy controls. In addition, patients with NIHSS score ≤3 had higher apelin-13 levels than those with NIHSS score >3. Low apelin-13 level in the patients was associated with death or major disability within 3-months, whereas patients with high apelin-13 levels showed a lower incidence of stroke and combined events after 1-year. These findings indicated that serum apelin-13 is a potential prognostic biomarker for acute ischemic stroke (Wang et al., 2020). Another clinical study demonstrated that higher apelin levels were associated with increased risk of stroke (including ischemic and hemorrhagic stroke) (Yu et al., 2021). Intravenous thrombolytic therapy (ITT) is commonly used to treat acute ischemic stroke, although it can enhance the risk of hemorrhagic transformation (HT). To analyze the predictive significance of apelin on HT in acute ischemic stroke patients after ITT, Zhu et al. (2021) analyzed the data of 109 acute ischemic stroke patients that received ITT, and found that a higher HT grade was associated with lower apelin level and increased levels of interleukin-1β (IL-1β) and IL-6. Moreover, lower apelin was also related with a higher risk of death of patients with both ischemic stroke and HT, indicating that apelin is an independent protective factor in stroke patients. Atrial fibrillation (AF) is associated with a high risk of stroke, and should therefore be detected in a timely manner. Bohm et al. (2021) showed that apelin levels were significantly lower in stroke patients with AF compared to the non-AF group in a multicenter, matched-cohort, and only apelin was identified as an independent predictor of AF. Thus, apelin administration should be considered in patients with high risk of stroke to exclude the possibility of AF. However, another clinical trial shown that apelin level did not differ between stroke patients and healthy individuals, and was not associated with cardiovascular mortality and morbidity during follow-up. This discrepancy can be attributed to differences in sample size and patients selection, and measurement assays for apelin. Further large-scale multicenter clinical trials are needed, along with detailed subgroup analysis, to clarify the therapeutic value of apelin in stroke.

### 3.2 Mechanistic investigation

#### 3.2.1 Apelin-13 protects against blood-brain barrier disruption after cerebral ischemia

The BBB controls the exchange of substances between blood and brain tissue, allows nutrients to pass and prevents harmful substances from entering, thereby protecting the CNS. Given that the secondary injuries after cerebral ischemia is closely related to the morphological and structural destruction of BBB, protecting the integrity of the latter and alleviating cerebral edema are increasingly being considered as treatment options for ischemic stroke (Huang et al., 2020; Parvez et al., 2022). Apelin-13 can reduce BBB permeability and brain vasogenic edema after ischemia by mitigating oxidative stress, and inhibiting the expression of matrix metalloproteinases (MMP) and endothelin-B receptor (Gholamzadeh et al., 2021a). Furthermore, the protective effect of apelin-13 on BBB post-stroke is significantly associated with the elevated expression of aquaporin-4 (AQP4), which is partly achieved by activating the extracellular signal-regulated kinase (ERK) and PI3K/Akt pathways (Chu et al., 2017). Several factors are involved in the destruction of BBB after ischemic stroke, such as inflammatory cytokines, microvessel and endothelial cell injury, and the degradation of extracellular matrix. It remains to be explored how apeline-13 affects these pathological pathways.

### 3.2.2 Apelin-13 promotes angiogenesis after cerebral ischemia

The apelin/APJ system plays an important role in embryonic vascular development and adult angiogenesis (Cox et al., 2006). Both APJ and apelin are expressed in retinal vascular endothelial cells, and apelin promotes the proliferation and chemotaxis of these cells, as well as formation of capillary tubes. In addition, the apelin/APJ system may also be involved in endothelial cell proliferation and neovascularization (Tao et al., 2010; Zhang et al., 2013). Apelin-13 can promote proliferation, migration and tube formation in myocardial microvascular endothelial cells, as well as angiogenesis *via* modulation of AMPK and Akt signaling (Yang et al., 2014a). Knocking out APJ in glioblastoma cells reduced tumor growth and angiogenesis, suggesting that targeting the apelin/APJ system is a promising strategy for preventing angiogenesis in glioblastoma (Amoozgar et al., 2019; Frisch et al., 2020).

Apelin-13 plays an important role in the formation of collateral circulation. A clinical trial demonstrated that apelin-13 was significantly increased in patients with moyamoya disease compared to those with middle cerebral artery occlusion independent of NO and VEGF. Given that moyamoya disease has better collateral circulation compared to ischemic stroke, high plasma levels of apelin may be indicative of good collateral circulation (Wu et al., 2022). Furthermore, intranasal administration of apelin-13 increased the number of new vessels in the area surrounding infarction, restored the local cerebral blood flow, and promoted long-term functional recovery by upregulating vascular endothelial growth factor (VEGF) and MMP-9 (Chen et al., 2015). Apelin-13 can protect neurovascular units from ischemic injury by increasing the expression of VEGF and VEGFR2, and promoting VEGF binding to VEGFR-2 by activating the ERK and PI3K/Akt pathways (Huang et al., 2016). Cerebral blood flow blockade is often accompanied by hypoxia, which activates the apelin/APJ system, and consequently promotes endothelial cell proliferation *via* the PI3K/Akt and MAPK signaling pathways (Zhang et al., 2015; Zhang et al., 2016).

### 3.2.3 Apelin-13 inhibits excitotoxicity after cerebral ischemia

Aspartic acid, glutamate and glycine are excitatory neurotransmitters that are mainly distributed in the synaptic terminals of neurons in the CNS. The main excitatory amino acid released after cerebral ischemia is glutamate, which binds to the excitatory amino acid receptors on the postsynaptic membrane, resulting in neurotoxicity and neuronal damage. Nerve cells are rich in NMDA, which mediates the excitotoxicity of glutamate. Glutamate activates NMDA receptors and triggers a massive Ca^2+^ influx through the specific ion channel, resulting in intracellular calcium overload in the early stage of ischemia, and eventually cell death (Hossmann, 1994; Lai et al., 2014). Apelin-13 can reduce NMDA activity by directly reducing the ion flow potential of the NMDA receptor membrane. In addition, apelin-13 also inhibits NMDA in a dose-dependent manner by activating the pro-survival Ca^2+^, IP3, PKC, MEK-1/2, Akt, and Raf/ERK-1/2 signaling pathways, thereby antagonizing the excitotoxicity effects of glutamate and alleviating neuron injury (Cook et al., 2011; O'Donnell et al., 2007). Another experimental study established that apelin protects against NMDA-induced retinal neuronal death *via* APJ receptor by activating Akt and ERK1/2, and downregulating TNF-α (Ishimaru et al., 2017). Zeng et al. (2010) showed that apelin-13 can prevent serum deprivation-induced changes in Akt and ERK1/2 phosphorylation, and attenuate NMDA-induced intracellular Ca^2+^ accumulation, which in turn inhibits apoptosis and excitotoxic death.

### 3.2.4 Apelin-13 promotes the stability of atherosclerotic plaques

Apelin-13 has been implicated in atherosclerosis in several studies on account of its immunoreactivity in human aortas and coronary arteries. Furthermore, apelin/APJ expression patterns are inversely correlated to human aortic and coronary atherosclerosis (Kostopoulos et al., 2014). In addition, serum apelin levels are negatively correlated with the severity of arterial stenosis, and positively correlated with the stability of atherosclerotic plaques, indicating its value as a potential biomarker of atherosclerotic plaque stability (Zhou et al., 2014). Consistent with this, a clinical trial conducted on 235 (114 black, 121 white) rheumatoid arthritis patients showed that apelin concentration in the serum was associated with altered levels of plaque stability mediators (MMP-2, MMP-9) and atherosclerosis, in a manner partly dependent on population origin and systemic inflammatory status (Gunter et al., 2017). A recent study showed that the apelin/APJ system is involved in the development of atherosclerosis by influencing vascular smooth muscle cells (Luo et al., 2018). Moreover, apelin is up-regulated in human atherosclerotic coronary artery and localized to the plaque along with macrophages and smooth muscle cells (Pitkin et al., 2010). Another study confirmed that apelin-13 significantly improves plaque stability by increasing collagen content and decreasing MMP-9 expression, reducing inflammatory cell infiltration (neutrophils and macrophages) and intracellular reactive oxygen species (ROS) content (Fraga-Silva et al., 2018). Furthermore, PINK1/Parkin-mediated mitophagy promotes apelin-13-induced vascular smooth muscle cell proliferation by AMPKα and exacerbates atherosclerotic lesions (He et al., 2019).

## 4 Summary and prospects

The apelin-13/APJ signaling axis is ubiquitous in the peripheral and central nervous systems. Apelin-13 is an endogenous neuroprotective molecule that regulates various physiological and pathological processes in the brain. Following cerebral ischemia, apelin-13 promotes angiogenesis, increases the stability of atherosclerotic plaques and reduces excitatory toxicity, thereby improving prognosis. At present, little is known regarding the function of the apelin-13/APJ pathway, and its mechanisms have not been clarified. To this end, we first need to clarify the biological functions and mechanism of apelin-13/APJ signaling in cerebral ischemia, and the long-term effects of activating this pathway. Secondly, novel APJ receptor agonists or antagonists have to be developed to verify the feasibility and efficacy of the apelin-13/APJ system as an intervention target in ischemic stroke. In addition, the variation loci related to the apelin/APJ system, their relationship with brain structure and function, and their impact on the prognosis of cerebral ischemia also need to be elucidated. Finally, the injection route, injection time and treatment frequency of apelin-13 in pre-clinical studies need to be optimized before clinical studies on ischemic stroke patients.
